# Evaluation of the Effects of Filler Fineness on the Properties of an Epoxy Asphalt Mixture

**DOI:** 10.3390/ma14082003

**Published:** 2021-04-16

**Authors:** Wei Xu, Jintao Wei, Zhengxiong Chen, Feng Wang, Jian Zhao

**Affiliations:** 1 School of Civil Engineering and Transportation, South China University of Technology, Guangzhou 510641, China; ctweijintao@mail.scut.edu.cn (J.W.); 201920107290@mail.scut.edu.cn (Z.C.); 202021010287@mail.scut.edu.cn (F.W.); 2 China Railway Construction Bridge Engineering Bureau Group Southern Engineering Co., Ltd., Guangzhou 511400, China; zhaojianll@126.com

**Keywords:** filler, epoxy asphalt, particle fineness, microscopic morphology, asphalt mixture, strength, experiment

## Abstract

The type and fineness of a filler significantly affect the performance of an asphalt mixture. There is a lack of specific research on the effects of filler fineness and dust from aggregates on the properties of epoxy asphalt (EA) mixtures. The effects of aggregate dust and mineral powder on the properties of an EA mixture were evaluated. These filler were tested to determine their fineness, specific surface area and mineral composition. The effects of these fillers on the EA mastic sample and mixture were evaluated. The morphology of the EA mastic samples was analyzed using scanning electron microscopy (SEM). The effects of the fillers on the Marshall stability, tensile strength and fatigue performance of the EA mixture were evaluated. The dust from the aggregates exhibited an even particle size distribution, and its average particle size was approximately 20% of that of the mineral powder. The SEM microanalysis showed that the EA mastic sample containing relatively fine dust formed a tight and dense interfacial bonding structure with the aggregate. The EA mixture sample containing filler composed of dust from aggregate had a significantly higher strength and longer fatigue life than that of the EA sample containing filler composed of mineral powder.

## 1. Introduction

The aggregate fraction of the asphalt mixture that passes through the 0.075 mm sieve is derived from aggregate dust and additional fillers (such as mineral powder). The dust contained in an aggregate was formed during the multistage aggregate crushing and grinding process. In comparison, conventional mineral powder is generally produced by ball milling, and the morphology and granularity of its particles are affected by the processing methods. For an asphalt mixture, the mass of its filler accounts for approximately 5–10% of its total mass, whereas the surface area of its filler accounts for a principal proportion of its total surface area [1]. The dust contained in aggregates at a certain proportion will significantly affect the properties of asphalt mixtures [2,3,4]. The effects of fillers on the properties of asphalt mixtures have been relatively extensively investigated. The type and fineness of a filler have been found to significantly affect the increase in the stiffness of an asphalt mixture [5]. The particle size analysis is a method to measure the distribution of various particle-size fractions of a filler [6]. Grabowski and Wilanowicz found that the particle size, specific surface area and particle texture of a filler significantly affected its capacity to enhance an asphalt mastic [7]. Antunes et al. [8] and Arulrajah et al. [9] studied the effects of the physical, geometric and chemical properties of fillers on the viscosity and antistripping of asphalt mastics and found that the fillers differ relatively significantly in particle size, geometric properties and surface characteristics, and that the physical and geometric properties of fillers relatively significantly affect the viscosity and antistripping performance of asphalt mastics, whereas their chemical composition has a relatively nonsignificant impact. Available studies have demonstrated that the properties and type of fillers significantly affect the strength, crack resistance and moisture susceptibility of asphalt mixtures [10,11,12,13,14].

Epoxy asphalt (EA) is a thermosetting polymer material consisting of a mixture of epoxy resin and asphalt [15,16]. An EA mixture is produced by mixing an EA binder, an aggregate and a filler. EA mixtures, which are high in strength and resistant to fatigue and corrosion compared to ordinary asphalt mixtures, are a typical type of pavement material for steel box girder bridge decks [17,18]. EA mixtures have thermosetting properties, and their basic properties differ considerably from those of ordinary asphalt mixtures [19]. 

The aggregate and filler requirements for EA mixtures are generally based on those for ordinary asphalt mixtures and are not designed for or specific to EA mixtures. Engineering practice often requires that the cleanliness of aggregates be improved and that the proportion of dust smaller than 0.075 mm in diameter in aggregates be reduced. As a result, particles smaller than 0.075 mm in diameter primarily come from mineral powder. However, there is a lack of evaluation of and basis for the reasonableness of these requirements for EA mixtures, and their impact on EA mixtures. From engineering tests on pavement mixtures for steel bridge decks, the authors have noticed that the proportion of dust smaller than 0.075 mm in diameter in aggregates will significantly affect the strength properties of EA mixtures, but the mechanism of this phenomenon is not clear. The evaluation of the effects of fillers on the properties of EA mixtures has yet to be reported to the best of the authors’ knowledge.

Therefore, it is necessary to systematically analyze both the effects of filler on the properties of EA mixtures and the mechanism of this influence. This study examined the effects of fillers on the properties of an EA mastic and a mixture. (1) The filler’ fineness distribution and specific surface area of dust contained in aggregates and mineral powder were determined; (2) The mineral composition of the fillers was determined; (3) The viscoelasticity of EA mastic samples containing fillers with different compositions of mineral powder and dust was evaluated through tensile and dynamic shear tests; (4) The morphology of filler particles and the characteristics of the fracture surfaces of the EA mastic samples were observed via scanning electron microscopy (SEM); (5) The properties of EA mixture samples containing fillers of different composition were evaluated through Marshall, tensile and fatigue tests. This study revealed the effects and mechanism of influence filler particle size and aggregate dust on the properties of an EA mixture and will provide a reference for the optimization of EA mixtures.

To clarify the terminology used in this study, epoxy asphalt (EA) refers to the mix of epoxy resin and asphalt without any filler, EA mastic refers to EA with filler, and EA mixture refers to EA with both filler and aggregate.

## 2. Materials and Methods

### 2.1. Materials

#### 2.1.1. Filler and Aggregate

Three aggregates, namely, diabase, basalt and limestone from a local aggregate plant (Zhongshan Aggregate Plant) in Guangdong Province, China, were selected and subsequently rinsed in water and sieved through a 0.075 mm sieve. The dust from each aggregate passing the 0.075 mm mesh was used as a filler and called aggregate dust. Additionally, two conventional limestone powder samples (A and B, designated mineral powder) were also selected and called mineral powder, and the fraction of each sample passing the 0.075 mm mesh was used as a comparative filler. A diabase aggregate was selected to prepare the EA mixture samples.

#### 2.1.2. EA

KD-BEP hot mix EA, produced by Kindai Kasei Corporation (Aichi-gun, Japan) with Shell A-70 base asphalt at a ratio of 28 (epoxy resin):22 (curing agent):50 (asphalt), was used in the test. Table 1 shows the performance parameters of KD hot mix EA.

#### 2.1.3. EA Mixture

Table 2 summarizes the EA mixture gradation from the test [20]. The optimal epoxy asphalt/aggregate ratio was 6.5%. The diabase dust was selected as the filler, and three schemes of filler composition were designed to evaluate its effects on the properties of the EA mixture: (A): the dust from aggregate was used as the particle fraction smaller than 0.075 mm in diameter; (B) the mineral powder was used as the particle fraction smaller than 0.075 mm in diameter; and (C) a mixture of dust (50%) and mineral powder (50%) was used as the particle fraction smaller than 0.075 mm in diameter.

#### 2.1.4. EA Mastics

EA mastic samples (filler/epoxy asphalt mass ratio: 1.85) were prepared based on the EA-10 mixture gradation shown in Table 2 and an optimal epoxy asphalt/aggregate ratio of 6.5%.

### 2.2. Method

#### 2.2.1. Testing of the Particle Size, Specific Surface Area, Micromorphology and Mineral Composition of the Fillers

(1) Particle size analysis

A Horiba LA-960S laser scattering particle size distribution analyzer (manufactured by Horiba, Ltd., Kyoto, Japan; measurement range: 0.01–3000 μm) was used.

(2) Specific surface area analysis

An ASAP 2020 N fully automatic rapid specific surface area analyzer (Micromeritics Instruments Corporation, Norcross, GA, USA) was used to measure the specific surface area of the fillers.

(3) Micromorphological observation

An S-3700N scanning electron microscope (Hitachi, Ltd., Tokyo, Japan) was used to observe the morphology of the filler particles.

(4) Mineral composition of the fillers

A D8 ADVANCE X-ray powder polycrystalline diffractometer (manufactured by Bruker Corporation, Berlin, Germany) was used to determine the mineral composition of the fillers.

#### 2.2.2. Tensile and Dynamic Shear Tests of EA Mastic Samples and SEM Micromorphological Observation of Their Tensile Fracture Surfaces

Asphalt mastics have a significant impact on the properties of asphalt mixtures [21,22]. The properties and microstructural morphology of the EA mastic samples containing various fillers were evaluated.

(1) Tensile test

Tensile tests were performed using an MTS 810 universal material tester (MTS Systems Co., Eden Prairie, MN, USA) at a strain rate of 500 mm/min at 23 °C, according to the American Society for Testing and Materials (ASTM) D638-14 [23].

(2) Microscopic observation of the fracture surfaces of tensile test samples

The morphology of the fracture surfaces of tensile test samples was observed via SEM. Additionally, the morphology of the fracture surface of the EA was compared after removing the asphalt by etching.

(3) Dynamic shear test of EA mastic samples

**Figure 1:**
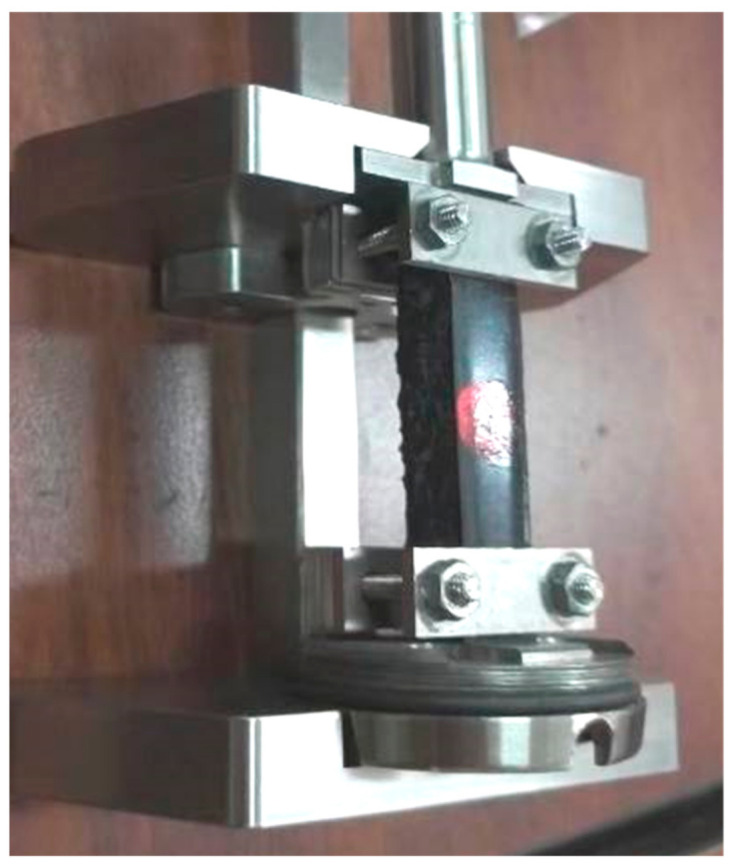
Dynamic shear test sample and fixture.

According to the ASTM D7552-09 [24], the viscoelasticity of the EA was evaluated based on the complex shear modulus (G*) and phase angle (δ) [25,26]. Rectangular prismatic EA mastic samples (50 mm × 12 mm × 10 mm) were prepared, as shown in Figure 1. Dynamic shear tests were conducted on the diabase dust-containing the EA mastic sample, the mineral powder A-containing EA mastic sample and pure EA sample at a scanning frequency of 0.1–10 Hz, a temperature of 60 °C and a strain level of 0.1%. An advanced Rheometer (AR), AR 2000 was used to test the specimens.

#### 2.2.3. Marshall, Tensile and Fatigue Tests of EA Mixture Samples

The effects of fillers on the properties of EA mixture samples were evaluated through Marshall, tensile and fatigue tests.

(1) Marshall stability test

According to the ASTM D6927-15 [27], the Marshall stability and flow values of EA mixture samples were determined.

(2) Tensile test of EA mixture samples

**Figure 2:**
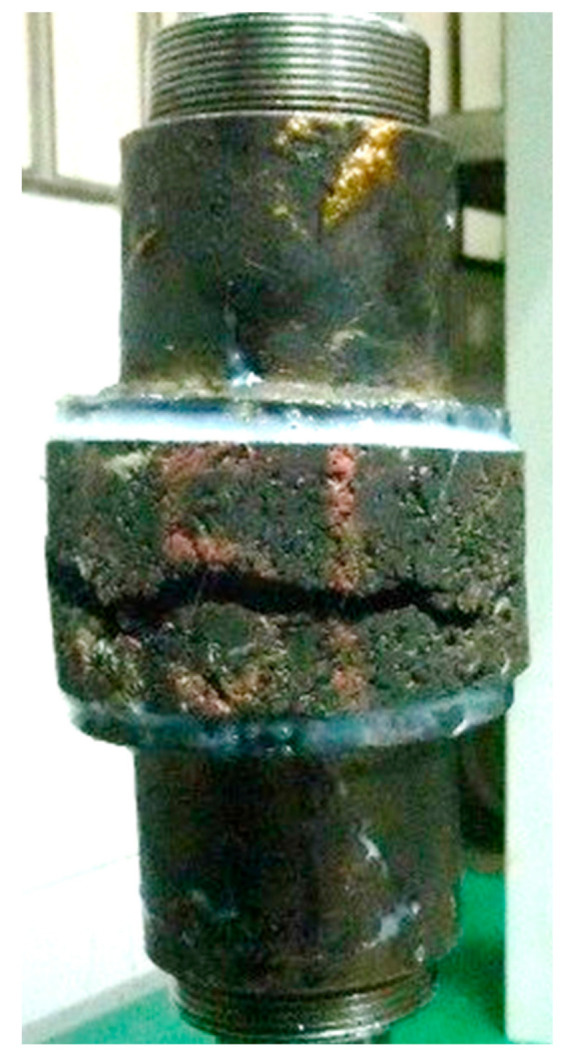
Tensile sample and pulling heads.

Tensile tests were performed to determine the tensile strength of the EA mixture samples. The pulling heads were bonded to each EA mixture sample, as shown in Figure 2. The Marshall EA mixture samples containing various fillers were each subjected to a tensile test on a universal testing machine at a tensile loading rate of 50 mm/min and a temperature of 23 °C.

(3) Fatigue test

EA mixture samples were subjected to a four-point bending fatigue test assessed according to American Association of State Highway Transportation Officials (AASHTO) T 321-07 at a temperature of 15 °C and a loading frequency of 10 Hz in the strain control mode [28]. The EA mixture sample exhibited an endurance limit at a strain level lower than 800 με, and its modulus basically became stable upon reducing to 60–70%, after which there was no significant change in its fatigue properties. On this basis, the differences in properties between EA mixture samples containing diabase dust and mineral powder A were evaluated at the strain levels of 1000 and 1250 με, respectively.

## 3. Results

### 3.1. Analysis of the Particle Size, Specific Surface Area, Particle Micromorphology and Mineral Composition of the Fillers

**Figure 3:**
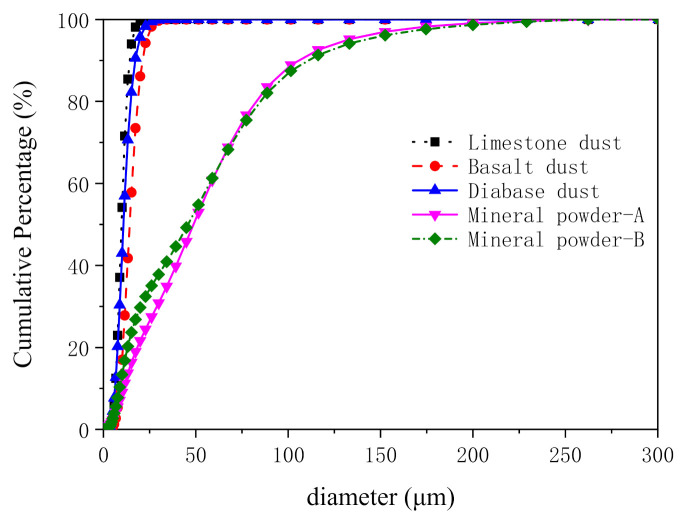
Particle size distribution curves of various fillers.

Figure 3 shows the particle size test results for the three types of dust contained in the aggregates and the two types of mineral powder. The particle size curves in Figure 3 show that dust particles were finer than the mineral powder particles and that there were notably more particles smaller than 25 μm in diameter in the three types of dust than there were in the two types of mineral powder. Table 3 summarizes the particle size analysis data for the fillers. As demonstrated by the data in Table 3, the three types of dust possessed relatively similar particle sizes, which were distributed relatively evenly. Additionally, for the three types of dust, the D_90_ content was 2.46 times that of the D_10_ content. The two types of mineral powder had relatively similar particle sizes, which were unevenly distributed. For the two types of mineral powder, the D_90_ content was 11.3 times that of the D_10_ content. The average particle size of the dust was approximately 20% that of the mineral powder.

(2) Specific surface area analysis

**Figure 4:**
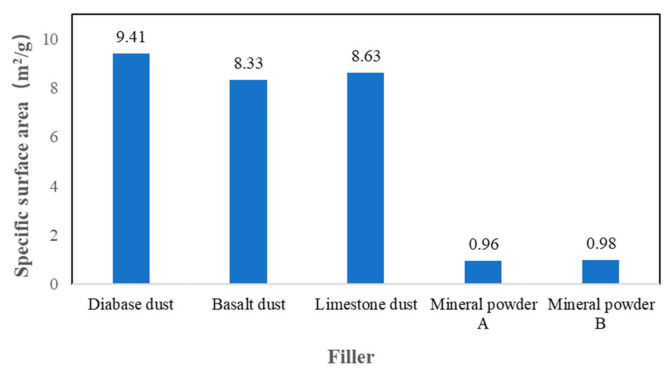
Specific surface area measurements of the fillers.

Figure 4 shows the specific surface area measurements for the diabase dust, basalt dust and limestone dust and for the two types of mineral powder. The average specific surface areas of the diabase dust, basalt dust and limestone dust were on average 9.70, 8.59 and 8.90 times that of the two types of comparative mineral powder, respectively. Clearly, the three types of dust each had a significantly higher specific surface area than that of the two types of mineral powder.

(3) Micromorphology of particles

**Figure 5:**
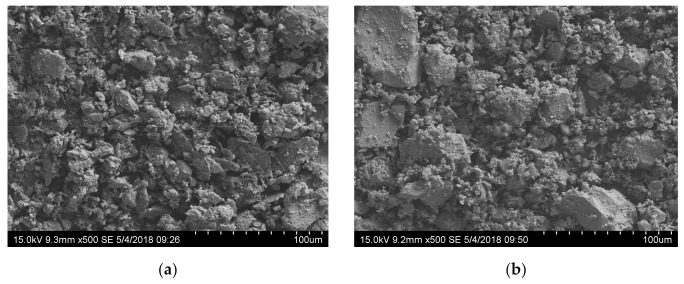
SEM images of filler particles: (**a**) diabase dust; and (**b**) mineral powder A.

The characteristics of the filler particles were relatively noticeable in the 500× magnification SEM images compared with other magnifications. The particles of the three types of dust exhibited similar morphologies. Figure 5a shows the SEM image of the diabase dust, which was representative of the three types of dust. Figure 5b shows the SEM image of the particles of mineral powder A, which was representative of the two types of mineral powder. As shown in the SEM images, the diabase dust particles exhibited relatively even particle sizes; in contrast, the mineral powder particles presented sizes that differed relatively significantly and were not evenly distributed.

(4) Mineral composition of the fillers

**Figure 6:**
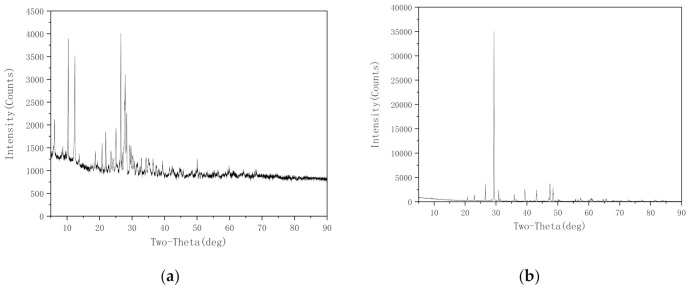
XRD patterns of the composition of fillers: (**a**) diabase dust; and (**b**) mineral powder-A.

Figure 6 shows the X-ray diffraction (XRD) patterns of the composition of the fillers. The three types of dust yielded similar XRD patterns. Diabase dust was selected as a representative sample, and its XRD pattern is shown in Figure 6a. As demonstrated in Figure 6a, the diabase dust contained a relatively large number of minerals, including SiO_2_, Na[AlSi_3_O_8_]-Ca[Al_2_Si_2_O_8_] and CaCO_3_. The two types of mineral powder were similar in composition and contained a relatively small number of minerals, mainly CaCO_3_ and CaMg[CO_3_]_2_. It is believed that the inorganic components of each filler would basically not undergo a chemical reaction with EA.

### 3.2. Analysis of the Effects of Fillers on the Properties of the EA Mastic

The effects of fillers on the tensile and viscoelastic properties of the EA mastic were evaluated.

#### 3.2.1. Test Analysis of Tensile Properties

**Figure 7:**
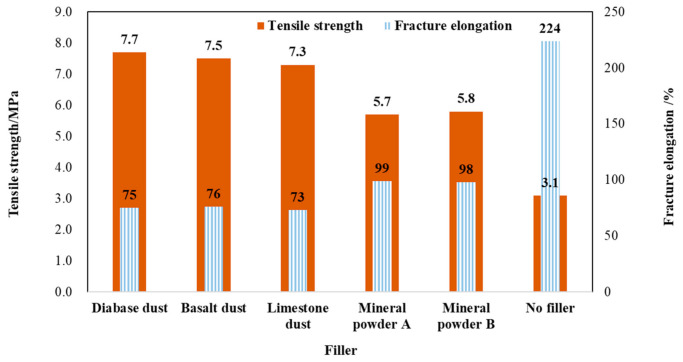
Tensile strength and elongation at the break of mastic (25 °C).

Figure 7 shows the tensile test data for the EA mastic samples. The data demonstrate that the fillers significantly enhanced the tensile strength of the EA mastic. Specifically, on average, the dust-containing EA mastic samples possessed a tensile strength approximately 30% higher than that of the mineral-powder-containing EA mastic samples. The dust-containing EA mastic samples presented an elongation at break approximately 25% lower than that of the mineral-powder-containing EA mastic samples. However, both the dust-containing and mineral-powder-containing EA mastic samples exhibited a relatively high elongation at break (>70%). The three types of dust differing in lithology yielded basically similar tensile strength values, suggesting that fineness was the primary factor affecting the tensile strength of the EA mastic and that dust lithology had no significant impact on the tensile strength of the EA mastic. As the production process is the same, compared with the normal mineral powder, the particle sizes of different types of dust in the project are similar and finer. The experiments show that the difference in fineness modulus between the powders and the dust is significant. Considering that the dust lithology showed no significant impact on the tensile properties of the EA mastic and that the three types of dust displayed similar particle size distributions, only the diabase dust, which is commonly used in steel bridge deck epoxy asphalt pavements, was used in the subsequent comparative tests between the EA mastic and mixture.

#### 3.2.2. Dynamic Shear Test Analysis of the EA Mastic

**Figure 8:**
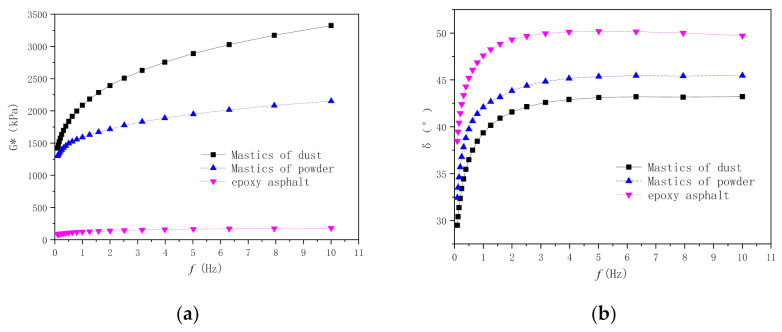
Viscoelastic properties of mastic samples at different frequencies (60 °C): (**a**) G*; and (**b**) δ.

**Figure 9:**
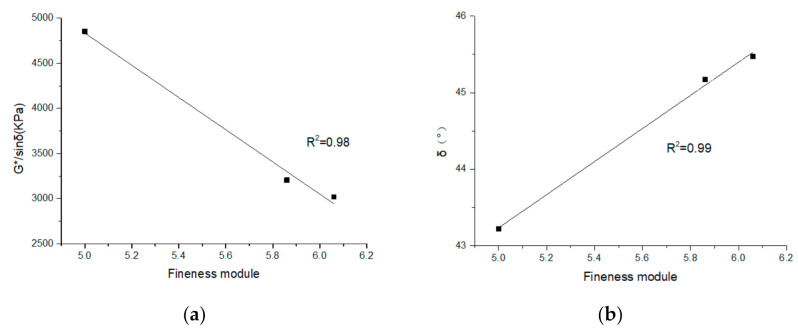
Analysis of the correlation between the fineness of the filler and the viscoelastic properties of the EA mastic (10 Hz): (**a**) G*/sinδ; and (**b**) phase angle (δ).

(1) Figure 8 shows the test curve data. As demonstrated in Figure 8, the complex shear modulus (G*) of the mastic increased significantly after adding a filler; however, the relatively fine dust more significantly increased G*. Additionally, the phase angle (δ) decreased substantially after adding a filler, suggesting that the filler significantly improved the elasticity of the mastic; similarly, the relatively fine filler more significantly improved the elasticity of the mastic.

(2) The rutting factor (G*/sinδ) and δ of the EA mastic samples at 60 °C and 10 Hz and the fineness modulus of the fillers were subjected to statistical regression analysis. Figure 9a shows that the G*/sinδ of the EA mastic decreased linearly as the fineness modulus increased, indicating that the relatively fine filler significantly improved the rutting factor of the EA mastic. Figure 9b shows that the δ of the mastic increased linearly with the fineness modulus (an empirical quantity obtained by adding the total percentage of the sample of a filler retained on each of a specified series of sieves, and dividing the sum by 100), indicating that the relatively fine filler considerably improved the elasticity of the EA mastic.

#### 3.2.3. SEM Observation of the Fracture Surface of Tensile Test Samples

**Figure 10:**
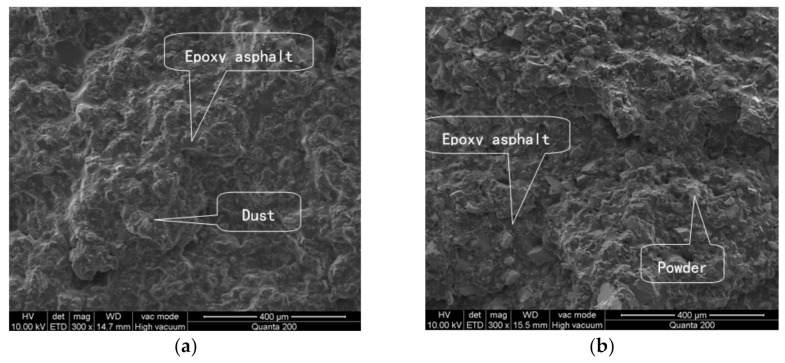
Comparison of the fracture surfaces of the mastic samples containing different fillers before etching: (**a**) diabase dust mastic; and (**b**) mineral powder mastic.

**Figure 11:**
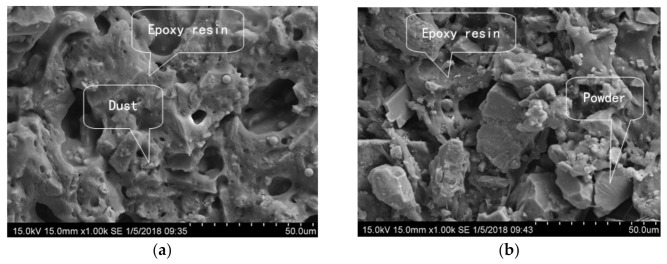
Comparison of the fracture surfaces of the mastic samples containing different dust fillers after etching: (**a**) diabase dust mastic; and (**b**) mineral powder mastic.

The fracture surface of each EA mastic sample fractured after the tensile test was etched using trichloroethylene to remove the asphalt and was subsequently examined using SEM to determine the micromorphology. The mechanisms by which the filler affected the adhesive properties of the EA and the strength of the EA mastic were analyzed. Figure 10 and Figure 11 (before and after etching, respectively) show the SEM images of the fracture surfaces of the mastic sample containing diabase dust and the comparative sample containing mineral powder A. The analysis results are provided below.

(1) Before etching, the fracture surface of each of the two mastic samples was level and smooth, and the filler was bonded tightly to the EA in each sample; no significant difference between the two mastic samples was apparent. After etching, the asphalt was removed by dissolution and the remaining filler of each sample was interlocked inside the spatial network structure formed after the epoxy resin solidified. The fracture surface of the dust-containing mastic sample was more intact than that of the mineral-powder-containing mastic sample. In the dust-containing mastic sample, the dust particles were relatively tightly and densely bound to the resin. In contrast, the fracture surface of the mineral-powder-containing mastic sample was relatively rough, with a relatively large number of voids between the mineral powder particles and the epoxy resin along with some exposed filler particles.

(2) The dust and EA formed a relatively tight bonding network, whereas a relatively large number of microdefects in the bonding network existed between the mineral powder and the EA. The relatively fine dust helped improve the compactness of the network structure of the EA mastic.

### 3.3. Analysis of the Effects of Fillers on the Properties of the EA Mixture

#### 3.3.1. Marshall Test Analysis

**Figure 12:**
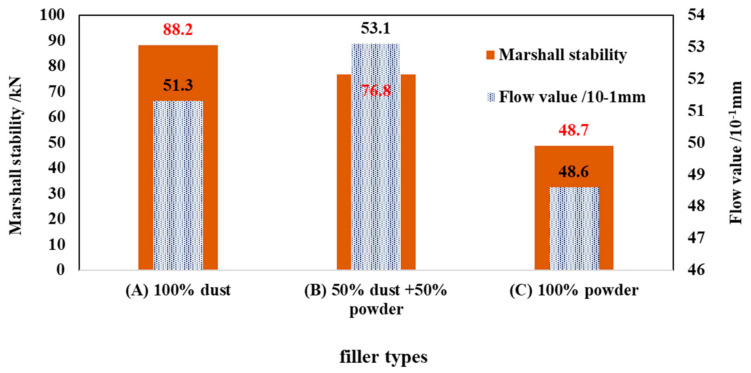
Results of the Marshall stability test.

Figure 12 shows the Marshall test results for the EA mixture samples. The results demonstrated that the EA mixture sample prepared based on scheme (A) yielded the highest Marshall stability, followed by those prepared based on scheme (B) and scheme (C). Specifically, on average, the EA mixture sample prepared based on scheme (A) revealed a 73% higher Marshall stability than that prepared based on scheme (B). Additionally, there was no notable difference in the flow value between the EA mixture samples prepared based on schemes (A) and (B), suggesting that, compared with the mineral powder, the relatively fine dust significantly enhanced the strength of the EA mixture.

#### 3.3.2. Tensile Test of the EA Mixture Samples

The tensile strength was calculated using Equation (1):

(1) $$\sigma =\frac{4F}{\pi \times D^{2}}$$

where σ is the tensile strength (MPa), *F* is the maximum pulling force applied to the pulling heads (kN), and *D* is the cross-sectional diameter (mm).

Table 4 summarizes the tensile test results for the EA mixture samples. The EA mixture sample prepared using scheme (A) presented the highest tensile strength, followed by those prepared using schemes (C) and (B), suggesting that the relatively fine dust significantly enhanced the tensile strength of the EA mixture, when compared with the effect of the mineral powder.

#### 3.3.3. Fatigue Test

The sample prepared based on scheme (A) revealed a fatigue life of 2.432 million and 299,000 cycles at 1000 and 1250 με, respectively, when its bending modulus was reduced to 50%. However, the sample prepared based on scheme (B) had a fatigue life of 645,000 and 46,000 cycles at 1000 and 1250 με, respectively, when its bending modulus was reduced to 50%. Compared with the mineral powder filler, the fatigue test data show that the relatively fine dust could significantly prolong the bending fatigue life of the EA mixture. This result is mainly because the relatively fine dust formed a tighter microstructural network with the EA, thereby enhancing the comprehensive strength and fatigue resistance of the EA mixture.

## 4. Discussion

The experimental data analysis shows that due to the thermosetting and higher strength characteristics of the EA mixture compared with ordinary asphalt mixtures, the filler fineness has a significant effect on the performance of the EA mastic and mixture. The fine particle dust contained in the aggregate is beneficial to the strength and deformation properties of the EA mixture. A relatively fine filler can enhance the strength properties of an EA mixture. On this basis, EA mixtures can be optimally designed to enhance the strength and antifatigue performance.

(1) As the production process is the same, compared with the normal mineral powder, the particle sizes of different types of dust in the project are similar and finer. The average particle size of the dust in each aggregate was approximately 20% that of the mineral powder. The particle sizes of the dust contained in each aggregate were evenly distributed, whereas the mineral particle sizes differed relatively significantly. The specific surface area of the dust in each aggregate was approximately nine times that of the mineral powder.

(2) The SEM microanalysis shows that the EA mastic samples containing relatively fine dust formed a tighter and denser interfacial bonding structure, which significantly improved the compactness of the network structure of the EA mastic and reduced its internal microdefects.

(3) The relatively fine fillers enhanced the EA mastic and mixture. The EA mastic samples containing relatively fine dust yielded a tensile strength approximately 30% higher than those containing mineral powder. Taking as a baseline the EA mixture samples containing pure mineral powder as the filler, the EA mixture sample containing relatively fine dust as the filler presented a 73% higher Marshall stability, a 158% higher tensile strength and an approximately four times longer fatigue life at control strain levels of 1000 and 1250 με.

(4) The lithology of dust from aggregate had no significant impact on the tensile strength of the EA mastic.

The quantitative evaluation of the effects and operating mechanism of fillers on more fine-grained value points, as well as the impact of a wider fineness range on the performance of EA mixtures, need further study to evaluate whether there is a critical point of fineness above which there is an impact on the respective mastic tensile properties.

## Figures and Tables

**Table 1:** Performance parameters of KD hot mix EA.

Property	Value	Test Method
Penetration (25 °C, 100 g, and 5 s), 0.1 mm	12.7	JTG E20-2011 T0604-2011
Softening point (ring and ball), °C	>100	JTG E20-2011 T0606-2011
Ductility (15 °C and 5 cm/min), cm	>200	JTG E20-2011 T0605-2011
Density (15 °C), g/cm^3^	1.01	JTG E20-2011 T0603-2011
Tensile strength (23 °C), MPa	3.46	ASTM D638
Elongation at break (23 °C), %	194	ASTM D638

**Table 2:** EA-10 mixture gradation.

Sieve Size (mm)	13.2	9.5	4.75	2.36	1.18	0.6	0.3	0.15	0.075
**Reference Range (%)**	100	95–100	65–85	50–70	39–55	28–40	21–32	14–23	7–14
**Percent Passing (%)**	100	99.5	75.6	54.8	41.2	33.1	22.9	18.4	12.0

**Table 3:** Particle size analysis data for the fillers.

Filler Types	Diabase Dust	Basalt Dust	Limestone Dust	Mineral Powder-A	Mineral Powder-B
Fineness Modulus	3.57	3.97	3.44	6.06	5.86
D_av_ (μm)	11.38	14.66	10.07	55.08	53.87
D_10_ (μm)	6.25	8.86	6.37	10.73	8.66
D_50_ (μm)	10.81	14.2	9.77	48.67	45.79
D_90_ (μm)	17.21	21.23	14.23	105.65	110.88

Notes: D_10_—particle diameter corresponding to 10% finer on the cumulative particle–size distribution curve; D_50_—particle diameter corresponding to 50% finer on the cumulative particle–size distribution curve (also referred to as the median particle diameter); D_90_—particle diameter corresponding to 90% finer on the cumulative particle–size distribution curve; Dav—average particle diameter.

**Table 4:** Tensile test results for the mixture samples (60 °C).

Sample	Filler Composition	Tensile Strength/MPa
Scheme (A)	100% dust	2.41
Scheme (B)	100% mineral powder	0.93
Scheme (C)	50% dust + 50% mineral powder	1.33

## Data Availability

Data sharing is not applicable to this article.
